# Opportunities for antibiotic stewardship in emergency department or hospitalized patients with asymptomatic bacteriuria: identifying risk factors for antibiotic treatment

**DOI:** 10.1017/ash.2022.4

**Published:** 2022-01-31

**Authors:** Morgan L. Bixby, Brian R. Raux, Aakansha Bhalla, Christopher McCoy, Elizabeth B. Hirsch

**Affiliations:** 1 University of Minnesota College of Pharmacy, Minneapolis, Minnesota; 2 Northeastern University, Boston, Massachusetts; 3 Medical University of South Carolina, Charleston, South Carolina; 4 Department of Pharmacy, Brigham and Women’s Hospital, Boston, Massachusetts; 5 Department of Pharmacy, Beth Israel Deaconess Medical Center, Boston, Massachusetts

## Abstract

Antibiotic treatment of asymptomatic bacteriuria (ASB) is considered inappropriate and may lead to adverse events. This 2-center, retrospective cohort study including emergency department or inpatient adults identified pyuria (odds ratio, 2.43; 95% confidence interval, 1.17–5.01; *P* = .02) as the only independent risk factor for antibiotic treatment of ASB.

Asymptomatic bacteriuria (ASB) occurs in the absence of urinary-specific symptoms such as frequency, urgency, dysuria, suprapubic tenderness, or costovertebral angle pain.^1^ Collection of urine cultures in asymptomatic patients can occur for various reasons and may be related to incorrect urine collection technique or as result of an ‘abnormal’ urinalysis ordered for noninfectious diseases workup, subsequently triggering automatic and unnecessary urine cultures.^2^ Infectious Diseases Society of America (IDSA) guidelines recommend against screening and treatment of ASB, in most cases with the exception of patients who are pregnant or are undergoing endourological procedures.^3^ Outside these 2 situations, antibiotic treatment of ASB is considered inappropriate and may lead to adverse events and increased antimicrobial resistance.^4^

Stewardship studies focused on reducing urine culture orders highlight lack of knowledge of indications for ordering cultures. One approach stewardship programs could take is targeted review of patients at highest risk of antibiotic treatment of ASB. In this study, we sought to identify characteristics associated with antibiotic treatment of ASB in both emergency department (ED) and hospitalized inpatients for the purposes of stewardship intervention design for reducing treatment of ASB.

## Methods

This 2-center, retrospective cohort study included unique adult, nonpregnant patients with consecutive nonduplicate monomicrobial urine isolates of Enterobacterales or *Pseudomonas aeruginosa* collected between August 2013 and January 2014 from a previous urine isolate susceptibility study.^5^ Patients were seen in either the emergency department (ED) or were admitted to 1 of 2 teaching hospitals in Boston, Massachusetts. The study was approved by the institutional review board of each hospital.

ASB was defined as patients without chart-documented urinary-specific symptoms of frequency, urgency, dysuria, suprapubic tenderness, costovertebral angle pain/tenderness, or purulent urethral drainage or discharge. Patients with fever (≥1 documented temperature of ≥38°C or 100.4°F during admission) or altered mental status in absence of urinary-specific symptoms were also considered to have ASB since isolated fever may be poorly indicative of UTI. Patient data were collected retrospectively from electronic medical records using a standardized data collection form which included demographics, urinary complicating factors, bacteriuria details, microbiologic data, and antibiotic usage. Pyuria was defined as >10 white blood cells per high-power field.

The primary outcome of the study was identification of variables associated with treatment of ASB with at least 1 dose of empiric antibiotics for presumed UTI. Statistical data were processed using R version 3.6.2 software and R Studio Desktop version 1.2.5042 software (R Studio, Boston, MA). Patient characteristics were compared using the Fisher exact for binary characteristics, the Welsh 2-sample *t* test for continuous, and the Kruskal-Wallis test for discrete numerical characteristics. Using a generalized linear model, univariate and multivariate logistic regression was performed using 5 patient characteristics with *P* < .05 in Table 1, clinically significant variables of confusion or altered mental status, and use of urinary catheter.

**Table 1.** Comparison of Patient Characteristics by Treatment of Empiric Antibiotics for a Presumed Urinary Tract Infection (UTI)

Characteristic	ASB Treated (*n* = 205)	ASB Untreated (*n* = 49)	*P* Value
Age, mean (SD)	70.1 (16.1)	70.7 (18.0)	.83
Sex, female, no. (%)	146 (71.2)	35 (71.4)	1.00
**Presenting location, no. (%)**			
Home	129 (62.9)	33 (67.3)	.62
Skilled nursing facility/long-term care facility	31 (15.1)	9 (18.4)	.66
Outside hospital	43 (21.0)	7 (14.3)	.33
Rehabilitation facility	1 (0.5)	0 (0.0)	1.00
**Hospital setting, no. (%)**			
Inpatient	109 (53.2)	26 (53.1)	1.00
Emergency department	96 (46.8)	23 (46.9)	1.00
**ICU patient, no. (%)**	68 (33.2)	14 (28.6)	.61
**APACHE II score, median (IQR)**	10.0 (7.0)	9.0 (5.0)	.59
**Length of stay, median d (IQR)**	6.0 (9.0)	4.0 (7.0)	.94
**Comorbidities, no. (%)**			
Cardiovascular disease	164 (80.0)	33 (67.3)	.08
Diabetes mellitus	73 (35.6)	15 (30.6)	.62
Hematological/oncological conditions	65 (31.7)	14 (28.6)	.73
Respiratory diseases	47 (22.9)	11 (22.4)	1.00
Anemia	31 (15.1)	7 (14.3)	1.00
Immunocompromising condition	33 (16.1)	6 (12.2)	.66
Renal disease	39 (19.0)	5 (10.2)	.21
Recurrent infection	19 (9.3)	1 (2.0)	.14
Inflammatory bowel disease	11 (5.4)	3 (6.1)	.74
Hepatic disease	13 (6.3)	2 (4.1)	.74
Peripheral vascular disease	15 (7.3)	2 (4.1)	.54
Transplant	7 (3.4)	0 (0.0)	.35
**Empiric antibiotic therapy, no. (%)**			
Ciprofloxacin	68 (33.2)	NA	NA
Cefepime	32 (15.6)	NA	NA
Trimethoprim/sulfamethoxazole	21 (10.2)	NA	NA
**Organism isolated, no. (%)**			
*Escherichia coli*	119 (58.0)	31 (63.3)	.52
*Klebsiella* spp	52 (25.4)	7 (14.3)	.13
*Pseudomonas aeruginosa*	18 (8.8)	7 (14.3)	.28
Other	17 (8.3)	4 (8.2)	1.00
**Urinalysis results^a^, no. (%)**			
Positive pyuria (> 10 WBC/high-power field)	161 (80.9)	26 (57.8)	<.01
Positive nitrites	103 (51.8)	16 (35.6)	.05
Positive casts	44 (22.1)	13 (28.9)	.33
**History of UTI, no. (%)**	42 (20.5)	6 (12.2)	.06
**Non–urinary-specific symptoms, no. (%)**			
Confusion or altered mental status	35 (17.0)	4 (8.2)	.18
Fever	29 (14.1)	1 (2.0)	.01
**Complicating factors, no. (%)**			
Ureteral stent	3 (1.5)	1 (2.0)	.58
Neurogenic bladder	5 (2.4)	0 (0.0)	.59
**Urinary catheter status, no. (%)**			
Presence of urinary catheter^b^	52 (25.4)	9 (18.4)	.36
Urinary catheter removed^c^	12 (5.9)	4 (8.2)	.52

Note. SD, standard deviation; ICU, intensive care unit; APACHE II, Acute Physiology and Chronic Health Evaluation; IQR, interquartile range; WBC, white blood cell count.

a Urinalysis was performed on 244 patient urine samples, 199 from the treated group and 45 from the untreated group.

b In place for >2 days and in place at the time of the positive urine culture.

c In place for >2 days and removed prior to the positive urine culture.

## Results

During the study, 449 patients with monomicrobial urine isolates were identified, and 254 met the inclusion criteria for ASB. Of those with ASB, 80.7% (n = 205) were treated with empiric antibiotics indicated for presumed UTI. Patients were balanced with regard to inpatient and ED settings (Table 1). Patients were primarily female, elderly (mean age, 70 years), and had at least 1 comorbidity.

The most commonly isolated urine organisms were *Escherichia coli* (59.1%), *Klebsiella* spp, and *Pseudomonas aeruginosa.* In 244 patients with urinalyses, positive pyuria and nitrites were significantly more frequent among treated patients. Non–urinary-specific symptoms of isolated fever and confusion or altered mental status both differed between groups, with fever being significantly more prevalent in treated patients. Complicating factors were uncommon; however, 30.3% of patients had catheter use for at least 2 days during their hospital stay.

During univariate logistic regression analysis, pyuria, nitrites, fever, and confusion or altered mental status were associated with treatment of ASB (*P* ≤ .20) and all but fever (due to the large confidence interval) were included in the multivariate model (Table 2). Even though univariate regression did not determine a urinary catheter use to be associated with ASB treatment, it was included in the multivariate regression model because it was thought to be a confounding variable important for adjustment.

**Table 2.** Logistic Regression Analyses for the Risk Factors for Empiric Antibiotic Treatment of ASB

Variable	Univariate Model		Multivariate Model^b^	
	Odds Ratio (95% CI)	*P* Value	Odds Ratio (95% CI)	*P* Value
**Non–urinary-specific symptoms**				
Fever	7.91 (1.63–142.70)	.04		
Confusion or altered mental status	2.32 (0.87–8.05)	.13	1.68 (0.60–5.95)	.36
**Urinalysis results**				
Positive pyuria	2.86 (1.41–5.72)	<.01	2.43 (1.17–5.01)	.02
Positive nitrites	1.90 (0.99–3.79)	.06	1.57 (0.78–3.54)	.20
**Urinary catheter^a^**	1.26 (0.64–2.61)	.52	1.08 (0.79–2.36)	.84

Note. ASB, asymptomatic bacteriuria.

a Any use of a urinary catheter during the hospitalization or emergency department visit.

b Receiver operating characteristic area under the curve = 0.65.

In the multivariate regression model, urinalysis results positive for pyuria (OR, 2.43; 95% CI, 1.17–5.01; *P* = .02) was the only independent risk factor associated with antibiotic treatment of ASB. Upon running a multivariate regression model considering possible variable interactions, none were shown to have any significant association with antibiotic treatment.

## Discussion

Inappropriate antibiotic treatment of ASB has been linked to significant risk for adverse events.^6^ A meta-analysis evaluating 5 clinical studies found no significant risk of ASB patients developing symptomatic UTI without treatment.^6^

In this retrospective, 2-center cohort study of 254 patients with ASB, 80.7% of patients received UTI-specific empiric antibiotic treatment for ASB. Most patients were elderly women with one or more comorbidity. Urinalysis positive for pyuria (OR, 2.43; 95% CI, 1.17–5.01; *P* = .02) was the only significant risk factor for treatment based on multivariate regression analysis. Although pyuria is often detected in patients with ASB, it has a low positive predictive value for identifying clinical infection and other studies have reported it to be a driving factor for antibiotic prescribing.^7,8^ Although both ED and hospitalized inpatients were included in our study, hospital setting did not contribute to inappropriate antibiotic treatment of ASB.

These results are comparable to 2 previous retrospective studies that evaluated risk factors for treatment of ASB.^9,10^ Both studies included only hospitalized inpatients in contrast to ours, which also included ED patients. In the first study of patients from 46 hospitals, positive urinalysis was also associated with treatment of ASB along with altered mental status, dementia, and more.^9^ This study also differed from ours in that urinalysis results were not separated. Despite the considerably larger sample size and more recent inclusion dates of 2016–2018, the proportion (82.7%) of patients with ASB treated with antibiotics was nearly identical. In a second study of ASB patients during a similar inclusion period as ours, urinalysis positive for nitrites and leukocyte esterase as well as *E. coli* isolate were shown to be significantly associated with ASB treatment.^10^ However, that study had a lower proportion of patients receiving UTI-specific antibiotic treatment (38%).

The retrospective design of this study is one of its limitations; we cannot guarantee all patient characteristics were recorded. Conversely, those with fever or altered mental status in absence of other urinary-specific symptoms were also defined as having ASB. Our data are also limited by the inclusion period (2013–2014); we hypothesize that rates of ASB treatment may already be decreased following recent educational campaigns. Although data were collected from 2 centers, our sample size was still relatively small, which may result in less generalizability of our findings. Finally, patients were primarily elderly, postmenopausal females; therefore, risk factors may differ for younger patients.

In summary, our results showed a large proportion of ASB patients were inappropriately treated with antibiotics. Efforts to curb future ASB treatment could involve urine diagnostic stewardship and creation of algorithms to flag patients with bacteriuria prescribed UTI antibiotics plus other institution-specific risk factors. This study suggests that elevated rates of antibiotic prescription for ASB warrants additional initiatives and we advocate for institution-specific stewardship alerts for focused review of patients at highest risk of inappropriate ASB treatment.
